# Single-cell sequencing of tumour infiltrating T cells efficiently identifies tumour-specific T cell receptors based on the T cell activation score

**DOI:** 10.1007/s00262-024-03710-9

**Published:** 2024-05-10

**Authors:** Chaoting Zhang, Shance Li, Luyan Shen, Xia Teng, Yefei Xiao, Wenjun Yang, Zheming Lu

**Affiliations:** 1 Key Laboratory of Carcinogenesis and Translational Research (Ministry of Education/Beijing), Laboratory of Biochemistry and Molecular Biology, Peking University Cancer Hospital & Institute, Beijing, 100142 China; 2 The Department of Pathology, the First Affiliated Hospital, Hainan Medical University, Haikou, 570102 China; 3 Key Laboratory of Tropical Translational Medicine of Ministry of Education, School of Basic Medicine and Life Sciences, Hainan Medical University, Haikou, 571199 China

**Keywords:** T cell receptor, Tumour infiltrating lymphocytes, Single-cell RNA sequencing

## Abstract

Adoptively transferred T cell receptor-engineered T cells are a promising cancer treatment strategy, and the identification of tumour-specific TCRs is essential. Previous studies reported that tumour-reactive T cells and TCRs could be isolated based on the expression of activation markers. However, since T cells with different cell states could not respond uniformly to activation but show a heterogeneous expression profile of activation and effector molecules, isolation of tumour-reactive T cells based on single activation or effector molecules could result in the absence of tumour-reactive T cells; thus, combinations of multiple activation and effector molecules could improve the efficiency of isolating tumour-specific TCRs. We enrolled two patients with lung adenocarcinoma and obtained their tumour infiltrating lymphocytes (TILs) and autologous tumour cells (ATCs). TILs were cocultured with the corresponding ATCs for 12 h and subjected to single-cell RNA sequencing. First, we identified three TCRs with the highest expression levels of IFNG and TNFRSF9 mRNA for each patient, yet only the top one or two recognized the corresponding ATCs in each patient. Next, we defined the activation score based on normalized expression levels of IFNG, IL2, TNF, IL2RA, CD69, TNFRSF9, GZMB, GZMA, GZMK, and PRF1 mRNA for each T cell and then identified three TCRs with the highest activation score for each patient. We found that all three TCRs in each patient could specifically identify corresponding ATCs. In conclusion, we established an efficient approach to isolate tumour-reactive TCRs based on combinations of multiple activation and effector molecules through single-cell RNA sequencing.

**Supplementary Information:**

The online version contains supplementary material available at 10.1007/s00262-024-03710-9.

## Introduction

Adoptive transfer of T cells genetically modified with T cell receptor (TCR) has demonstrated effective antitumour activities and provided new possibilities in cancer therapy [1, 2]. Tumour-reactive TCRs, which are essential for the antitumour response of TCR-engineered T cells (TCR-T), can be identified from T lymphocytes stimulated and activated by tumour antigens. Neoantigens arising from somatic mutations are capable of activating and enriching tumour-specific T cells that demonstrated antitumour efficacy [3–7]. However, the number of neoantigens per patient with immunogenicity in epithelial cancer is small [8]; moreover, accumulating evidence suggested that many tumour-reactive antigens were derived from noncoding regions [9], indicating a desperate need to screen tumour-specific antigens, especially for cancer patients with low mutation burdens.

Tumour antigens expressed by autologous tumour cells (ATCs) are unbiased stimulators in eliciting tumour-specific T cell response [10–13]. When T cells are activated by corresponding tumour antigens, they express surface activation markers such as 41BB, CD69 and CD25 and produce effector molecules such as IL-2, TNF-*α*, IFN-*γ*, perforin and granzymes [14, 15]. Therefore, previous studies reported that tumour-reactive T cells were isolated based on the expression of the surface activation markers (41BB) [13] or the production of effector molecules (IFN-*γ*) [16]. However, T cells could not uniformly respond to activation but rather showed a heterogeneous expression profile of activation markers and effector molecules [17, 18]. This could derive from oscillating and stochastic expression of activation markers and effector molecules in T cell developmental processes [19]. In addition, it could be due to intrinsic difference of effector responses to antigens in different T cell types. Thus, isolation of tumour-reactive T cells based on a single activation marker or effector molecules could result in the absence of tumour-reactive T cells, and combinations of multiple activation markers and effector molecules could improve the efficiency of isolating tumour-reactive T cells and corresponding TCRs.

Although a small fraction of tumour-infiltrating T cells (TILs) could specifically identify tumour antigens, the effector function of these TILs suffering from the immunosuppressive tumour microenvironment could be impaired, which could further result in decreased expression of activation markers and cytotoxic effector molecules in tumour-reactive TILs stimulated by corresponding tumour antigens [20]. Therefore, it is urgently needed to establish a sensitive and efficient method to obtain dysfunctional tumour-reactive T cells with lower levels of activation marker expression and effector molecule secretion. High-throughput single-cell RNA sequencing (scRNA-seq) and single-cell TCR sequencing (scTCR-seq) provide a powerful tool to define TCR sequences and gene expression profiles for each T cell and could be used to efficiently screen tumour-reactive T cells and corresponding TCRs according to differentially expressed gene analysis.

In this study, we attempted to isolate tumour-specific TCRs through combination analysis of multiple activation markers and effector molecules in TILs activated by corresponding ATCs through high-throughput scRNA-seq and scTCR-seq. Using this strategy, we developed a sensitive and efficient procedure to generate individualized tumour-reactive TCRs and TCR-Ts for the treatment of solid tumours.

## Method and materials

### Patient samples

This project was approved by the Institutional Review Board of the Peking University School of Oncology, China. Two patients with lung adenocarcinoma provided written informed consent. Each tumour sample was collected in tubes containing sterile saline solution and cut into several fragments for a) TIL culture, b) generation of autologous tumour cells (ATCs), and c) generation of a patient-derived xenograft (PDX) model.

### Cell staining and flow cytometry

Cells were stained with antibodies for flow cytometry, including Fixable Viability Stain 780(FVS780), BV510-conjugated anti-CD3(UCHT1), APC R700 anti-mTCR(H57-597), BV421 anti-CD137(4B4-1), APC anti-CD69(FN50) (BD Biosciences, Franklin Lakes, NJ, USA). For mTCR and other membrane molecules detection, cell staining steps were performed at room temperature for 15 min with 1 × PBS washes between steps.

### Generation of TILs, ATCs and PDX models as well as cell lines

TILs were generated as previously described [13]. Briefly, each tumour sample was cut into 1–2 mm fragments, and then each fragment was put into a well of a 24-well plate containing T cell media and 50 ng/mL OKT3 antibody (ACRO, USA). T cell media included X-VIVO 15 (Lonza, USA), IL-2 (50 U/mL, Perprotech, USA), IL-15 (10 ng/mL, Perprotech, USA), IL-7 (10 ng/mL, Perprotech, USA), and Glutamax (Life Technologies, USA). TILs were cultured in a 37 ℃ incubator with 5% CO_2_ and passaged until there were enough TILs used for screening tumour-reactive T cells.

Tumour fragments mixed with Matrigel were subcutaneously implanted into immunodeficient NOD-SCID mice to establish a PDX model in accordance with our previous study [13]. Autologous tumour cells were obtained from a successfully established PDX model [13]. In brief, tumour specimens from PDX were cut into small fragments and put into GentleMACS tubes. The tumour fragments were mechanically dissociated into single-cell suspensions using a gentleMACS Octo Dissociator with Heaters (Miltenyi Biotech, Germany) according to the manufacturer’s instructions. Single-cell suspensions were washed with 1 × phosphate-buffered saline (PBS), run through a 100 μM cell strainer and then resuspended in 6-well plates with complete media (DMEM, 20% FBS, 2 mM GlutaMAX) at 37 °C with 5% CO_2_.

To produce high-titer lentivirus supernatants, HEK 293-FT (Life Technologies) originally purchased from the American Type Culture Collection (Manassas, Virginia, USA) was used as a packaging cell line, which was cultured in complete Dulbecco’s modified Eagle’s medium (DMEM, Gibco, USA) supplemented with 10% fetal bovine serum (FBS, Gibco, USA) containing 0.1 mM MEM nonessential amino acids, 1 mM sodium pyruvate and 2 mM GlutaMAX (Life Technologies, USA) at 37 °C with 5% CO_2_.

### Single-cell RNA-seq and TCR-seq capturing, library construction and sequencing

Single-cell capturing and library construction were carried out using Chromium Single Cell 3 g Reagent kits v.2 and kit v.3 (10 × Genomics) as well as Chromium Single Cell V(D)J Reagent kits (10xGenomics) for TILs according to the manufacturer’s protocol. In brief, single-cell suspensions, barcoded gel beads and partitioning oil were loaded onto Chromium Chip A/B to obtain single-cell gel bead-in-emulsions. Then, the polyadenylated transcripts were reverse-transcribed in each gel bead-in-emulsion. Full-length cDNA along with cell barcode identifiers were amplified by PCR, and sequencing libraries were prepared and normalized afterwards. The constructed library was sequenced on an Illumina NovaSeq 6000 S4.

### Data preprocessing and analysis of single-cell RNA-seq and TCR-seq

The Cell Ranger Software Suite (v.3.0.1) was used to demultiplex cellular barcodes, map reads to the Homo_sapiens.GRCh38.93.chr genome and transcriptome using splicing-aware aligner STAR [21] and process unique molecular identifier (UMI) counting. Seurat (v4.0.1) was used to analyse most of the single-cell RNA-seq data. The following criteria were applied to each cell of two patients: gene number between 800 and 10,000, mitochondrial gene percentage < 0.2, and the average UMI count of CD3D, CD3E and CD3G > 0. After applying these criteria, a total of 6831 and 8321 cells were left for the following analysis for patient 1 and patient 2, respectively.

TCR V(D)J segments were enriched from amplified cDNA from 5′ libraries using a Chromium Single-Cell V(D)J Enrichment kit according to the manufacturer’s protocol (10 × Genomics). TCR sequences for each single T cell were assembled by Cell Ranger vdj pipeline (v.2.2) to identify CDR3 sequences and the rearranged TCR genes. Cells with both productive TCR *α*- and *β*-chains were kept, and for cells with more than one *α*- or *β*-chain, the chain with higher UMIs or reads was kept. After applying these criteria of the abovementioned single-cell TCR-seq, a total of 6403 and 8004 cells were left for the following analysis for patient 1 and patient 2, respectively. T cells were assigned to be the same T cell clone if they had both the same *α* and *β* chains based on the nucleotide sequence.

In summary, after applying the abovementioned criteria of both single-cell RNA-seq and TCR-seq, a total of 6144 and 7818 cells were finally left for the following analysis for patient 1 and patient 2, respectively.

### Dimensionality reduction and clustering

The filtered gene-barcode matrix was normalized using ‘LogNormalize’ methods in Seurat (v.4.0.1) with default parameters. To exclude the influence of TCR V and J genes on clusters, TCR V and J genes were not used for clustering analysis. The top 2000 variable genes were detected using the ‘vst’ method in the Seurat FindVariableFeatures function. The variables ‘nCount_RNA’ and ‘percent.mito’ were regressed out in the scaling step, and principal component analysis (PCA) was performed using the top 2000 variable genes. Then, UMAP was used to visualize the cells on the top 20 principal components. Graph-based clustering was performed based on the PCA-reduced data for clustering analysis using Seurat (v.4.0.1). Functional annotations for clusters were manually curated using a set of differentially expressed genes for each cluster and visual inspection of canonical markers using UMAP visualization.

### Differential gene expression analysis

The FindAllMarkers function in Seurat (v.4.0.1) was used for differential gene expression analysis. For each cluster, differentially expressed genes (DEGs) were generated relative to all the other cells. A gene was considered significant with avg_log2FC > 0.25 and pct.1 > 0.1. The DEG results are summarized in Table S1.

### The calculations of T cell activation scores

T cell activation scores were calculated using the Seurat AddModuleScore function [21], which considers a core list of 10 genes associated with T cell activation (IFNG, IL2, TNF, IL2RA, CD69, TNFRSF9, GZMB, GZMA, GZMK, and PRF1).

### Construction of lentivirus vectors and transduction of T cells

TCR*α*/*β* nucleotide sequences were synthesized (GenScript) and cloned into our lentivirus backbone [13]. The insertion sequence was codon optimized for expression in human tissues. TCRs were constructed in a *β*–*α* chain order and their constant regions were exchanged by mouse counterparts modified with added disulfide bond and hydrophobic substitution as previously delineated, which not only was used to identify TCR-T, but improved TCR/CD3 stability and TCR pairing [13, 22]. To generate TCR-T, T cells were transduced with lentivirus as previously delineated [13]. In brief, peripheral blood mononuclear cells (PBMCs) were isolated and cultured in T cell media supplemented with 50 ng/ml OKT3 and 1 μg/ml anti-CD28 for 2 days before transduction. TCR lentivirus were generated by cotransfection of 293-FT cells with both TCR lentivector and packaging plasmids using PEI MAX 40,000 (Polysciences Inc. USA) [13]. T cells were transduced by lentivirus with 8 μg/mL polybrene (Sigma–Aldrich, USA) and 2 days later, the transduction efficiency was asessed using mouse TCR-*β* chain constant region staining through flow cytometry.

### IFN-γ ELISA

A total of 1 × 10^6^ effector T cells were incubated with 1 × 10^5^ ATCs in the absence of exogenous cytokines for 18–24 h. Coculture supernatant was transferred into a new 96-well plate, and IFN-*γ* concentration was measured by using a commercially available human IFN-*γ* ELISA kit (ExCell Bio, China) according to the manufacturer’s protocols.

### In vitro* TCR-T cell cytotoxicity assay*

To evaluate cytotoxic activity, ATCs were prelabeled with carboxyfluorescein succinimidyl ester (CFSE, BD Biosciences), and then a total of 2 × 10^4^ ATCs were cocultured with T cells at room temperature for 4–6 h at E:T ratios of 2:1, 10:1 and 30:1. Following coincubation, 1 μg/mL propidium iodide (PI, BD Biosciences) was added to evaluate the ratio of cell death. BD Accuri C6 (BD Biosciences) was used to analyse the samples. The percentage of ATCs killing is calculated as the proportion of CFSE and PI double-positive cells to all CFSE-positive cells.

### Treatment of PDX models by TCR-Ts

NOD/SCID mice (Beijing Huafukang Animal Technology Co., Ltd.) were used to establish patient-derived xenografts approved by the Institutional Review Board of the Peking University School of Oncology, China. The tissues were obtained through surgical resection or biopsy and must be handled with utmost care to preserve its integrity and viability. Once harvested, the tissue is transported to the laboratory under strict conditions that maintain its sterility and physiological environment. And then tissues were minced into so small fragments (less than 1 mm × 1 mm) that could go through syringe needle and mixed well with an equal volume of matrix gel. The fragments are then implanted into the inguinal region of immunocompromised mice, such as NOD-SCID mice, which lack a functional immune system and thus allow the engraftment of human tumour cells. Each NOD/SCID mouse was implanted 150 μL tumour suspension. Tumour treatment was performed on day 3 following tumour cell injection and consisted of a single intravenous injection of 6 × 10^6^ T cells (either TCR-Ts or untransduced T cells). IL-2 was administered three times with a 2-day interval, followed by tumour monitoring. Tumour size was measured three times a week and calculated using the following formula: tumour volume (mm^3^) = [(length) × (width) × (width)]/2.

### Statistical analysis

Statistical analysis was performed using GraphPad Prism 7.0 (GraphPad Software, CA) and R (version 3.4.0). For differential expression calculations in single-cell gene expression data, the DECENT package implements a likelihood ratio test to determine statistical significance. For all differential expression and gene set testing analyses, *P* values were corrected for multiple testing using the Benjamini–Hochberg method. For other statistical analyses, statistical comparisons were performed with unpaired Student’s t test and one-way analysis of variance with Bonferroni post hoc tests. All tests were two-sided, and a *p* value < 0.05 was considered statistically significant.

### Data availability

The raw scRNA-seq and scTCR-seq data can be accessed in the Sequence Read Archive (BioProject No. PRJNA766698).

## Results

**Fig. 1:**
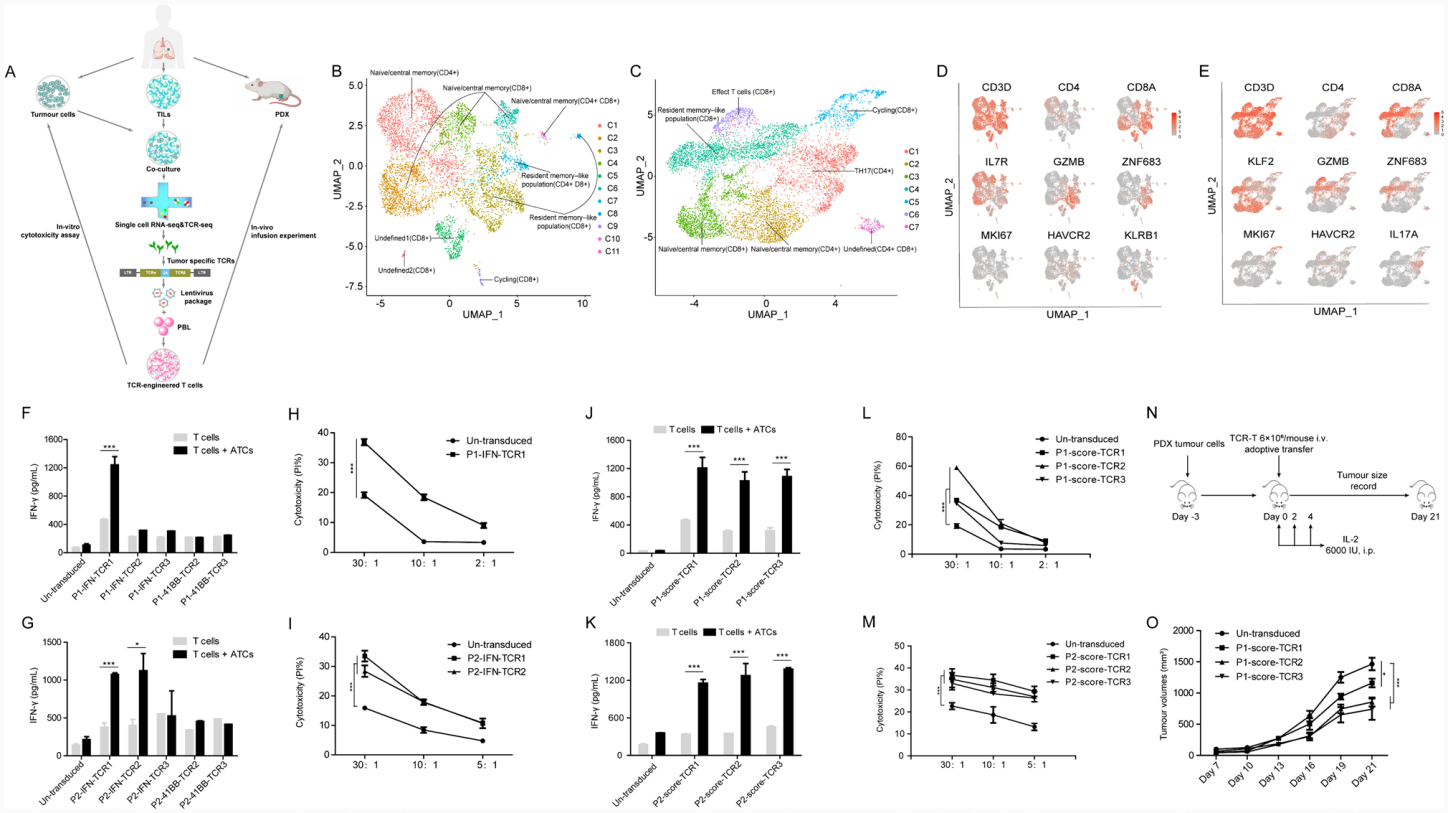
Identification of tumour-specific TCRs and functional verification of corresponding TCR-Ts. **A**, Flowchart for isolation of TCRs from TILs through single-cell RNA sequencing and functional verification of corresponding TCR-Ts. First, tumour specimens were resected from a patient with lung adenocarcinoma, and then TILs, patient-derived xenograft (PDX) models and autologous tumour cells (ATCs) were obtained. ATCs were cocultured with the corresponding TILs for 12 h, and subsequently, the stimulated TILs were subjected to scRNA-seq and scTCR-seq to identify candidate tumour-specific TCRs. Subsequently, the candidate tumour-reactive TCRs were cloned into lentiviral vectors and introduced into peripheral T cells to generate tumour-reactive TCR-Ts. Finally, we evaluated whether these TCR-Ts could specifically recognize and kill ATCs in vivo and in vitro. **B–C**, UMAP of scRNA-seq data from TILs in patient 1 (**B**) and in patient 2 (**C**). Clusters are denoted by colours and labelled with inferred cell states. **D–E**, Cells colored by normalized expression of the indicated markers on UMAP in patient 1 (**D**) and in patient 2 (**E**). **F–G**, TCRs identified by single-cell sequencing based on the expression levels of IFNG and TNFRSF9 in patient 1 (**F**) and patient 2 (**G**) were transduced into donor T cells, and then transduced T cells were cocultured with ATCs for each patient. The secretion of IFN-*γ* from T cells was determined by ELISA. Data are representative of at least three independent experiments with more than three different donors. **H–I**, Cytotoxicity capacity of tumour-reactive TCR-Ts against ATCs in patient 1 (**H**) and patient 2 (**I**). The line chart summarizes the cytotoxicity by subtracting the ATC spontaneous death at different E:T ratios. Data are representative of at least three independent experiments with more than three different donors. **J–K**, TCRs identified by single-cell sequencing based on the activation score in patient 1 (**J**) and patient 2 (**K**) were transduced into donor T cells, and then transduced T cells were cocultured with ATCs for each patient. The secretion of IFN-*γ* from T cells was determined by ELISA. Data are representative of at least three independent experiments with more than three different donors. **L–M**, Cytotoxicity capacity of tumour-reactive TCR-Ts against ATCs in patient 1 (**L**) and patient 2 (**M**). The line chart summarizes the cytotoxicity by subtracting the ATC spontaneous death at different E:T ratios. Data are representative of at least three independent experiments with more than three different donors. **N**, Schematic experimental plan. Tumour treatment was performed on day 3 following tumour cell injection and consisted of a single intravenous injection of T cells (either TCR-Ts or untransduced T cells). IL-2 was administered three times with a 2-day interval, followed by tumour monitoring. **O**, Antitumour activity of tumour-reactive TCR-Ts in patient 1 against patient-derived xenograft models. The tumour size is plotted on the y axis. Time after infusion of T cells is plotted on the x axis. The mean values from each group are plotted. Error bars represent the SEM (n = 6 mice per group, *p < 0.05, ***p < 0.001, analysed by one-way analysis of variance with Bonferroni posttest

The procedure of isolating tumour-specific TCRs is summarized in Fig. 1A. First, tumour specimens were resected from a patient with lung adenocarcinoma, and then TILs, autologous tumour cells (ATCs) and patient-derived xenograft (PDX) models were obtained, as previously delineated [13]. ATCs were cocultured with the corresponding TILs for 12 h, and subsequently, the stimulated TILs were used for scRNA-seq and scTCR-seq. The scRNA-seq was used to determine the expression level of a single T cell activation marker, such as IFN-*γ* or 41BB, as well as the combined expression levels of various T cell effect markers in each T cell, and scTCR-seq was used to identify TCR*α*/*β* sequences of corresponding T cells. To test this new approach, TILs (TIL1 and TIL2) isolated from two patients (patient 1 and patient 2) with lung adenocarcinoma were used in this study.

We primarily performed high-throughput scRNA-seq and scTCR-seq to efficiently identify tumour-specific T cells and TCRs according to the expression levels of activation and effector molecules. After a series of quality control filtering of scRNA-seq and scTCR-seq, a total of 6144 and 7818 eligible T cells were left for patient 1 and patient 2, respectively (Table S1). There were 447 and 1856 clonotypes, each having unique productive *α*–*β* chain pairs, out of which 204 and 612 were represented by two or more cells for patient 1 and patient 2, respectively (Table S2). Unbiased clustering was performed and visualized using UMAP (Fig. 1B–C). We identified one CD4^+^ T cell cluster, eight CD8^+^ T cell clusters, and two CD4^+^ CD8^+^ double-positive T cell clusters for patient 1 and two CD4^+^ T cell clusters, four CD8^+^ T cell clusters, and one CD4^+^ CD8^+^ double-positive T cell cluster for patient 2 (Fig. 1B–C). The differentially expressed functional markers indicated clusters of CD8^+^ T cells (naïve/central memory, resident memory, cycling and undefined), CD4^+^ T cells (naïve/central memory), and CD4^+^ CD8^+^ double-positive T cells (naïve/central memory and resident memory) for patient 1 and clusters of CD8^+^ T cells (naïve/central memory, resident memory, effect, and cycling), CD4^+^ T cells (naïve/central memory and TH17), and CD4^+^ CD8^+^ double-positive T cells (undefined) for patient 2 (Fig. 1B–E and Table S3). These clusters were named based on previously defined T cell states in multiple single-cell transcriptomic studies [23, 24].

Since tumour-specific T cells stimulated by corresponding tumour antigens could express activation markers, we attempted to identify tumour-specific T cells and TCRs on basis of the expression levels of activation molecules through high-throughput scRNA-seq and scTCR-seq. However, some tumour-nonspecific TILs could start signaling and express activation molecules, which could be due to the abundance of nontumour-specific stimuli or due to suitability to in vitro conditions. Theoretically, if one tumour-specific TCR could recognize corresponding ATCs, all T cells with this TCR should also be activated. Although heterogenicity of tumour cells existed, most TILs with the same tumour-reactive TCR should also be activated by ATCs. Thus, to eliminate the influence of nonspecific stimuli against TILs, we identified tumour-specific T cells and corresponding TCRs based on average levels of activation markers in all T cells with the same TCR clonotype. To reduce the bias, TCRs expressed on at least three T cells were included in the subsequent analysis. Previous studies reported that upregulation of IFN-*γ* (gene name, IFNG) and 41BB (gene name, TNFRSF9) expression on activated T cells could be applied to recognize and isolate tumour-specific T cells. Therefore, we first identified three TCRs (P1-IFN-TCR1, P1-IFN-TCR2 and P1-IFN-TCR3 for patient 1; P2-IFN-TCR1, P2-IFN-TCR2 and P2-IFN-TCR3 for patient 2) with the highest average expression levels of IFNG mRNA in all T cells with the same TCR clonotype for each patient (Table 1). Next, we also recognized three TCRs (P1-41BB-TCR1, P1-41BB-TCR2 and P1-41BB-TCR3 for patient 1; P2-41BB-TCR1, P2-41BB-TCR2 and P2-41BB-TCR3 for patient 2) with the highest average expression levels of TNFRSF9 mRNA in all T cells with the same TCR clonotype for each patient (Table 1).

**Table 1:** The characteristics of TCRs based on expression level of IFNG and TNFRSF9 as well as activation score

Patient	Group	Rank in each group	Rank in activation score group	Cell number of each TCR	TRAV	TRAJ	CDR3 of TCR α	TRBV	TRBD	TRBJ	CDR3 of TCR β	Nomalized expression of IFNG	Nomalized expression of TNFRSF9	Activation score
P1	IFN	1	3	3	TRAV1-2	TRAJ28	CAVRDQEEYSGAGSYQLTF	TRBV4-3	TRBD1	TRBJ1-6	CASSPGVGEYNSPLHF	1.74	1.08	0.74
P1	IFN	2	19	3	TRAV38-1	TRAJ39	CAFTYMNNNAGNMLTF	TRBV7-6	TRBD2	TRBJ2-1	CASSGPGLAGGEQFF	0.96	0.00	0.42
P1	IFN	3	56	3	TRAV21	TRAJ15	CAAPNQAGTALIF	TRBV7-9	TRBD2	TRBJ2-2	CASSATSGEAGELFF	0.84	0.00	0.09
P1	41BB	1	3	3	TRAV1-2	TRAJ28	CAVRDQEEYSGAGSYQLTF	TRBV4-3	TRBD1	TRBJ1-6	CASSPGVGEYNSPLHF	1.74	1.08	0.74
P1	41BB	2	86	3	TRAV12-2	TRAJ33	CAPMDSNYQLIW	TRBV18	TRBD2	TRBJ2-3	CASSPREGMADTQYF	0.00	0.32	0.00
P1	41BB	3	30	12	TRAV13-1	TRAJ36	CAATDQTGANNLFF	TRBV29-1	None	TRBJ2-1	CSVEVWENEQFF	0.35	0.22	0.30
P1	activation score	1	1	3	TRAV21	TRAJ23	CAGHGYNQGGKLIF	TRBV7-9	TRBD2	TRBJ2-5	CASSSTSGGPIQETQYF	0.25	0.00	0.79
P1	activation score	2	2	4	TRAV8-4	TRAJ45	CAVSGLSGGGADGLTF	TRBV27	None	TRBJ1-1	CASSLRVNTEAFF	0.00	0.00	0.77
P1	activation score	3	3	3	TRAV1-2	TRAJ28	CAVRDQEEYSGAGSYQLTF	TRBV4-3	TRBD1	TRBJ1-6	CASSPGVGEYNSPLHF	1.74	1.08	0.74
P2	IFN	1	5	3	TRAV12-1	TRAJ9	CVVYTGGFKTIF	TRBV20-1	TRBD1	TRBJ2-2	CSASLRGKRGTGELFF	1.94	0.56	0.67
P2	IFN	2	7	4	TRAV35	TRAJ38	CAAHAGNNRKLIW	TRBV7-3	TRBD1	TRBJ2-3	CASSLGGVDTQYF	1.25	0.00	0.60
P2	IFN	3	114	6	TRAV17	TRAJ30	CATVFNRDDKIIF	TRBV5-1	TRBD2	TRBJ1-1	CASSFWDLNTEAFF	1.17	0.00	0.15
P2	41BB	1	5	3	TRAV12-1	TRAJ9	CVVYTGGFKTIF	TRBV20-1	TRBD1	TRBJ2-2	CSASLRGKRGTGELFF	1.94	0.56	0.67
P2	41BB	2	84	7	TRAV12-1	TRAJ41	CVVTNSNSGYALNF	TRBV6-3	TRBD2	TRBJ2-3	CASRSGRRTDTQYF	0.55	0.50	0.21
P2	41BB	3	116	5	TRAV27	TRAJ50	CAGPMKTSYDKVIF	TRBV12-4	None	TRBJ2-7	CASSLNSYEQYF	0.50	0.39	0.15
P2	activation score	1	1	3	TRAV8-4	TRAJ6	CAVPRSGGSYIPTF	TRBV28	None	TRBJ2-7	CASSPIPLYGSHEQYF	0.42	0.00	0.83
P2	activation score	2	2	4	TRAV35	TRAJ35	CAGQEGGFGNVLHC	TRBV27	TRBD2	TRBJ1-2	CASSERALAGYTF	0.00	0.00	0.76
P2	activation score	3	3	6	TRAV41	TRAJ44	CAVDRGTASKLTF	TRBV9	TRBD1	TRBJ2-1	CASRGTGDEQFF	1.15	0.00	0.70

To test the reactivity of these TCRs, peripheral blood T cells were transduced with each TCR and cocultured with ATCs overnight. Since the TCR*α*/*β* chain sequences of P1-IFN-TCR1 and P1-41BB-TCR1 were the same and the TCR*α*/*β* chain sequences of P2-IFN-TCR1 and P2-41BB-TCR1 were also identical (Table 1), we evaluated the antitumour activity of five TCR-engineered T cells for each patient.

We found that only P1-IFN-TCR1-, P2-IFN-TCR1- and P2-IFN-TCR2-transduced T cells recognized the corresponding ATCs (Fig. 1F–G). In addition, these three TCR-Ts also showed cytotoxic activity against matched ATCs, as measured with a flow cytometry-based cytotoxicity assay (Fig. 1H–I). It is interesting to elucidate why of five candidate tumour-specific TCRs identified by single-cell RNA sequencing in each patient, only one or two TCRs could identify and kill corresponding ATCs for each patient. Although single-cell sequencing is a sensitive approach to detect low expression levels of mRNA, it could also result in false positive results, especially for lower expression levels of mRNA. In addition, T cells with different cell states could not uniformly respond to activation but rather exhibit a heterogeneous expression profile of activation markers and production profile of effector molecules [17, 18]. Thus, isolation of tumour-reactive T cells based on a single activation marker or effector molecule could also result in false positive and false negative results, and combinations of multiple activation markers and effector molecules could improve the accuracy and efficiency of isolating tumour-reactive T cells.

When T cells are activated, they are expected to express IFNG, IL2, TNF, IL2RA, CD69, TNFRSF9, GZMB, GZMA, GZMK, and PRF1. Although each activated T cell generally cannot express all the above activation markers and effector molecules due to T cell dysfunction or oscillating expression of activation markers, most activated T cells can express multiple activation markers and effector molecules. Therefore, we defined the T cell activation score based on the average expression of IFNG, IL2, TNF, IL2RA, CD69, TNFRSF9, GZMB, GZMA, GZMK, and PRF1 mRNA for each T cell and then identified three TCRs (P1-score-TCR1, P1-score-TCR2 and P1-score-TCR3 for patient 1; P2-score-TCR1, P2-score-TCR2 and P2-score-TCR3 for patient 2) with the highest activation score in all T cells with the same TCR clonotype for each patient (Table 1). Peripheral blood T cells were transduced with each TCR and cocultured with ATCs overnight. IFN-*γ* ELISA demonstrated that all six TCR-transduced T cells recognized the corresponding ATCs (Fig. 1J–K). The flow-cytometry results showed that expression levels of CD137 and CD69 significantly increased in TCR-T cells cocultured with corresponding autologous tumour cells (Supplemental Figure S1). In addition, all six TCR-T cells demonstrated cytotoxic activity against matched ATCs, as measured with a flow cytometry-based cytotoxicity assay (Fig. 1L–M). Next, all six TCR-T cells of two patients displayed in vitro identification and cytotoxicity against corresponding ATCs, suggesting the feasibility and effectiveness of this individualized tumour-specific TCR screening approach. To simplify the in vivo functional validation of tumour-specific TCR-T cells, we only assessed the therapeutic effect of P1-score-TCR1, P1-score-TCR2 and P1-score-TCR3-engineered T cells with patient-derived xenograft (PDX) models. The PDX model from patient 1 underwent adoptive infusion of untransduced T cells, P1-score-TCR1, P1-score-TCR2, and P1-score-TCR3-engineered T cells (Fig. 1N). P1-score-TCR1, P1-score-TCR2, or P1-score-TCR3-engineered T cells exhibited significantly higher in vivo antitumour activity than untransduced T cells (Fig. 1O).

## Discussion

In this study, we describe an efficient approach to identify and isolate tumour-reactive TCRs based on a combination of T cell activation markers and effector molecules (IFNG, IL2, TNF, IL2RA, CD69, TNFRSF9, GZMB, GZMA, GZMK, and PRF1) through single-cell sequencing. Since T cells with different cell states could not uniformly respond to activation but rather exhibit a heterogeneous expression profile of activation markers and production profile of effector molecules [17, 18], isolating tumour-reactive TCRs based on the mRNA level of a single activation molecule (IFNG or TNFRSF9) through single-cell sequencing could result in the absence of tumour-reactive T cells. Since most activated T cells express multiple activation markers and effector molecules, we found that combinations of multiple activation markers and effector molecules could improve the accuracy and efficiency of isolating tumour-reactive T cells over a single marker.

When T cells are stimulated and activated by corresponding antigens, they express surface activation markers such as 41BB, CD69 and CD25 and produce effector molecules such as IL-2, TNF-*α*, IFN-*γ*, perforin granzymes [14, 15]. Therefore, several studies reported that tumour-reactive T cells were isolated on the basis of the expression of surface activation markers (41BB) [13] or the production of effector molecules (IFN-*γ*) [16]. As expected, our study also found that TCRs of TILs with the highest average expression level of IFNG or TNFRSF9 mRNA using high-throughput RNA-seq could specifically recognize matched ATCs in each patient, suggesting that high-throughput RNA-seq provides a sensitive and efficient procedure to identify dysfunctional tumour-reactive T cells with lower levels of activation marker expression and effector molecule secretion. However, we identified three TCRs with the highest expression levels of IFNG and TNFRSF9 mRNA for each patient, yet only the top one or two TCRs recognized the corresponding ATCs in each patient. In addition, the top TCR with the highest expression levels of IFNG or TNFRSF9 mRNA was identical in each patient. These findings indicated that although single-cell sequencing is a sensitive approach to detect low expression levels of mRNA, isolation of tumour-reactive T cells based on the mRNA level of a single activation marker or effector molecule through single-cell sequencing could result in false positive results, especially for T cells expressing lower levels of activation markers or effector molecules. Since most activated T cells express multiple activation markers and effector molecules, we anticipated that combinations of multiple activation markers and effector molecules could improve the accuracy and efficiency of isolating tumour-reactive T cells over a single marker.

When T cells are activated by tumour antigens, they are expected to express IFNG, IL2, TNF, IL2RA, CD69, TNFRSF9, GZMB, GZMA, GZMK, and PRF1. Although each activated T cell generally cannot express all the above activation markers and effector molecules due to T cell dysfunction or oscillating expression of activation markers, most activated T cells can express multiple activation markers and effector molecules. Thus, we next asked whether combinations of activation markers and effector molecules could improve the accuracy and efficiency of isolating tumour-reactive T cells over a single marker. We defined the activation score based on normalized expression levels of IFNG, IL2, TNF, IL2RA, CD69, TNFRSF9, GZMB, GZMA, GZMK, and PRF1 mRNA for each T cell and then identified three TCRs with the highest activation score for each patient. We found that a total of six TCR-engineered T cells in two patients could specifically identify and kill corresponding ATCs. These findings indicated that isolation of tumour-reactive T cells based on a single activation marker or effector molecule could result in the absence of tumour-reactive T cells and that combinations of multiple activation markers and effector molecules could improve the efficiency of isolating tumour-reactive T cells over a single marker.

In conclusion, we established a sensitive and efficient approach to isolate tumour-reactive TCRs based on combinations of multiple activation markers and effector molecules through single-cell RNA-seq for basic and translational research as well as for clinical applications.

### Supplementary Information

Below is the link to the electronic supplementary material.

Supplementary file1 (XLSX 842 KB)

Supplementary file2 (XLSX 609 KB)

Supplementary file3 (XLSX 9964 KB)

## Data Availability

All data generated or analysed during this study are included in the manuscript, and all de-identified data generated or analyzed during this study are available upon request.
